# Reduced Coupling of Global Brain Activity and Cerebrospinal Fluid Flow in Individuals With Betel Quid Dependence

**DOI:** 10.1111/adb.70164

**Published:** 2026-05-10

**Authors:** Li Li Fu, Chao Qi Lv, Li Ting Liu, Qing Qing Fu, Wei Yuan Huang, Yi Hao Guo, Tao Liu, Hui Juan Chen, Feng Chen

**Affiliations:** ^1^ Department of Radiology Hainan General Hospital/Affiliated Hainan Hospital of Hainan Medical University Haikou Hainan China; ^2^ School of Information and Communication Engineering Hainan University Haikou Hainan China; ^3^ Department of Geriatric Center Hainan General Hospital/Affiliated Hainan Hospital of Hainan Medical University Haikou Hainan China

**Keywords:** addiction neuroimaging, betel quid dependence, gBOLD‐CSF coupling, glymphatic function, resting‐state fMRI

## Abstract

Millions of people worldwide are affected by betel quid dependence (BQD), a prevalent psychoactive substance‐use disorder. To evaluate cerebral glymphatic dysfunction in BQD, this work used the coupling between global blood‐oxygen‐level‐dependent (gBOLD) and cerebrospinal fluid (CSF) signals collected via resting‐state functional MRI (rs‐fMRI). Sixty‐six participants (29 BQD individuals and 37 healthy controls) underwent rs‐fMRI scanning. Cross‐correlation analysis between cortical BOLD and CSF time series was carried out to compute the gBOLD‐CSF coupling intensity. Group comparisons were performed via independent sample *t*‐tests, and correlations with clinical features were assessed using exploratory Pearson's analysis. Bonferroni correction was applied to the five correlation analyses (BQDS, duration, daily consumption, HAMA‐14 and HAMD‐24). The corrected significance threshold was set at *p* < 0.01 (0.05/5). BQD individuals exhibited significantly reduced gBOLD‐CSF coupling (*t* = −2.42, *p* = 0.019), and weaker coupling correlated with longer BQD duration (*r* = 0.4313, uncorrected *p* = 0.0195). After Bonferroni correction for multiple comparisons, the correlation between duration and gBOLD‐CSF coupling (original *p* = 0.0195) did not reach the adjusted threshold. Chronic betel quid exposure may impair glymphatic function, reflecting disrupted brain‐CSF interactions. The gBOLD‐CSF coupling metric could be used as a noninvasive imaging biomarker to evaluate neurotoxic effects and glymphatic dysfunction in substance‐use disorders.

## Graphical abstract

Betel quid dependence (BQD) is a prevalent psychoactive substance‐use disorder with unclear neuropathology. Using resting‐state fMRI, we evaluated cerebral glymphatic function in BQD individuals by measuring the coupling between global BOLD and cerebrospinal fluid signals. BQD patients showed significantly reduced gBOLD‐CSF coupling, which was associated with longer dependence duration, suggesting that chronic betel quid exposure may impair glymphatic function and that this metric could serve as a noninvasive imaging biomarker for neurotoxic effects.

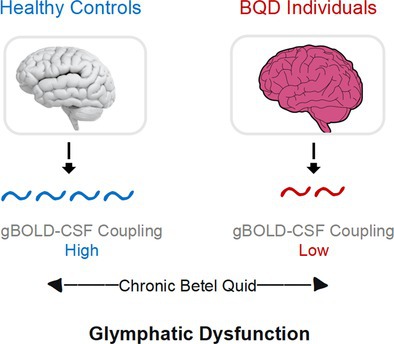

## 1 Introduction

Betel quid (BQ) consumption is an addictive and psychoactive behaviour. BQ ranks fourth in prevalence after the addictive triad of nicotine, alcohol and caffeine [1]. Approximately 600 million individuals worldwide regularly consume BQ [2]. Arecoline, the most significant physiological substance in BQ, markedly increases serotonin (5‐hydroxytryptamine, or 5‐HT) level, a chemical messenger that leads to pleasure, euphoria and serves as a contributing factor to betel quid dependence (BQD) [3]. Prolonged heavy consumption of BQ can lead to dependence and a spectrum of neuropsychiatric and oncological complications, including mood instability and oral malignant diseases [4, 5]. BQ's extensive use has been categorized as ‘addiction’ due to its neurochemical similarities to nicotine [6]. Despite the well‐documented clinical consequences, the underlying neurological mechanisms of BQD remain poorly understood.

The cerebral glymphatic system—responsible for clearing metabolic waste and neurotoxins such as amyloid‐β (Aβ)—plays a vital role in maintaining brain homeostasis [7, 8]. The majority of cerebrospinal fluid (CSF) migrates from the subarachnoid area into cerebral tissue via periarterial pathways. The subsequent interchange of CSF and interstitial fluid inside cerebral tissue comprises the glymphatic clearance process [9, 10]. Passage through the parenchyma is necessary for this exchange, and it is regulated by aquaporin‐4 (AQP‐4) water channels. In the end, this process enables the clearance of both CSF and metabolic waste products into the perivenous space [11]. As a key monoamine neurotransmitter and vasoactive substance, 5‐HT not only modulates cerebral vascular tone and neurovascular coupling via specific receptors [12, 13] but also plays a critical role in regulating sleep–wake cycles. Specifically, dorsal raphe 5‐HT neurons are most active during wakefulness and exhibit markedly reduced activity during nonrapid eye movement (NREM) sleep [14]. Because glymphatic transport depends in part on perivascular fluid movement driven by arterial pulsatility, 5‐HT‐mediated vascular changes may indirectly influence glymphatic inflow. Concurrently, glymphatic transport is known to be enhanced during slow‐wave sleep and suppressed during wakefulness [15]. Consequently, elevated 5‐HT levels could theoretically impair glymphatic function through dual pathways: by altering vascular dynamics related to arterial pulsatility and by disrupting sleep architecture. Arecoline, acting as a monoamine oxidase‐A inhibitor, increases 5‐HT levels. Therefore, arecoline‐induced serotonergic alterations may provide a plausible mechanistic link between BQ exposure and glymphatic dysfunction. A dysfunctional glymphatic system results in neurotoxic substance accumulation, which has been implicated in substance dependence. Liu Qiang et al. [16] conducted a mouse experiment demonstrating that alcohol exposure impairs glymphatic function and reduces the clearance rate of parenchymal Aβ, potentially contributing to cognitive decline and the onset of dementia. Furthermore, Lundgaard et al. [17] found that chronic consumption of ethanol can induce reactive neuroglia and adversely affect glymphatic function, which may elevate dementia risk among heavy drinkers. Additionally, Chen et al. [18] discovered that glymphatic system function in mice exposed to cocaine was significantly compromised, leading to impediments in the clearance of cerebral waste. These findings collectively suggest a connection between the pathogenesis of substance dependence and glymphatic dysregulation. Despite these insights, no study has examined glymphatic alterations in BQD.

Several established noninvasive MRI‐based biomarkers—including measurements of choroid plexus volume (CPV), diffusion tensor imaging along the perivascular space (DTI‐ALPS) and the coupling between global blood‐oxygen‐level‐dependent signals and cerebrospinal fluid signals (gBOLD‐CSF coupling)—have been used to quantify the function of the cerebral glymphatic system [19]. Among them, the gBOLD‐CSF coupling provides a sensitive proxy for cortical glymphatic activity by capturing synchronized low‐frequency oscillations between neural and CSF signals [20, 21, 22]. A decrease in gBOLD‐CSF coupling reflects impaired glymphatic function. Altered gBOLD‐CSF coupling has been reported across numerous neurological conditions, including Alzheimer's disease [23], Parkinson's disease [24] and the behavioural form of frontotemporal dementia [25]. Given the chronic neurotoxic exposure and vascular perturbations in BQD, we hypothesized that the gBOLD‐CSF coupling in individuals with BQD would be reduced, reflecting impaired glymphatic function.

## 2 Materials and Methods

### 2.1 Inclusion and Exclusion Criteria

The ethics review board at our hospital gave its approval for this study (Number 2017‐4). Prior to inclusion, each participant gives their informed consent. Betel Quid Dependence Scale (BQDS) for self‐report was applied to assess the use of BQ. Participants were considered to have BQD if their BQDS score exceeded 4. In our study cohort, all participants consumed a traditional preparation consisting of areca nut, slaked lime and *Piper betle* leaf, without tobacco or other additives.

The following requirements had to be met in order for BQD volunteers to be included: (1) They had to be between the ages of 18 and 60; (2) either non‐smokers or individuals who had used nicotine only occasionally (no more than once or twice per month during the previous 3 years); (3) addiction often co‐occurs with mental illnesses, especially affective (e.g., depression) and anxiety disorders; to eliminate potential influences of depression and anxiety, self‐report evaluations were conducted using the Hamilton Depression Rating Scale‐24 item (HAMD‐24) and the Hamilton Anxiety Rating Scale‐14 item (HAMA‐14); (4) BQD subjects were required to have a BQDS score greater than 4, with HAMD‐24 scores not exceeding 7 and HAMA‐14 scores not exceeding 7; (5) availability of complete images; and (6) right‐handedness.

The healthy controls (HCs) must fulfil the following requirements in order to be included: (1) be between the ages of 18 and 60; (2) have no prior usage of cigarettes, alcohol, areca nuts or BQ; (3) have availability of complete MRI data; and (4) be right‐handed.

Exclusion criteria for all subjects included the following: (1) abusing or using other addictive substances or drugs; (2) current or past history of alcohol and/or tobacco addiction; (3) presence or history of systemic disorders or psychosis and family history of mental illness; (4) contraindications to MRI assessment and any lesion or abnormal signals observed on MR images; and (5) left‐ or bilateral‐handed.

**FIGURE 1:**
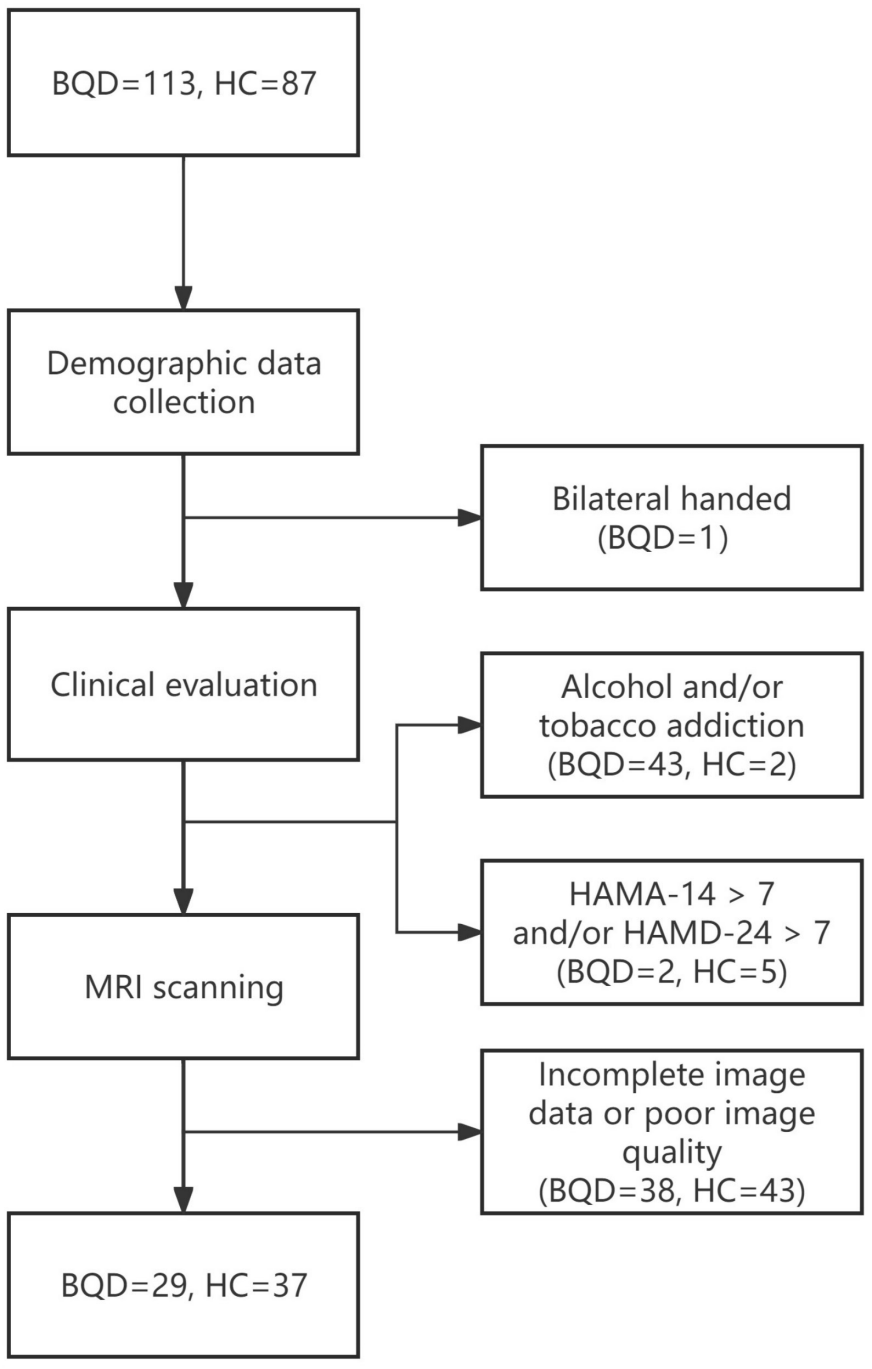
Participant's recruiting and exclusion process chart. Abbreviations: BQD, betel quid dependence; HAMA‐14, Hamilton Anxiety Rating Scale‐14 item; HAMD‐24, Hamilton Depression Rating Scale‐24 item; HC, healthy control.

Ultimately, a total of 29 BQD individuals and 37 controls were recruited from the local population in Hainan province. A schematic flowchart illustrating participant recruitment and exclusion procedures is shown in Figure 1.

### 2.2 Questionnaire

All participants were evaluated using a comprehensive questionnaire, which collected demographic and clinical data including age, gender, educational level, daily BQ dosage, duration of BQD and tobacco and alcohol consumption. BQDS was employed to evaluate the degree of BQD. The BQDS has demonstrated good internal consistency (α = 0.92) and construct validity [26], and it is currently the most widely used tool for assessing the state of BQD. On the day of scanning, we utilized the HAMD‐24 and the HAMA‐14 to evaluate levels of depressive and anxious symptoms among participants. Alcohol Use Disorders Identification Test (AUDIT) and Fagerstrom Test for Nicotine Dependence (FTND) were used to assess the alcohol and tobacco addiction status in this study.

### 2.3 MRI Data Acquisition

MRI data were acquired using a Siemens Skyra 3.0‐T scanner equipped with a 32‐channel head coil. Each person involved was instructed to maintain a stationary position of their heads within an MRI device, keep their eyes open but not focus on anything and utilize foam headrests to minimize any potential rotation of the head. The whole‐brain BOLD signal was taken by a gradient‐echo echo‐planar imaging (GRE‐EPI) sequence. Detailed parameters were as follows: repetition time (TR) = 2000 ms, echo time (TE) = 30 ms, field of view (FOV) = 224 × 224 mm^2^, image matrix = 64 × 64 and slice thickness = 3.5 mm. There were 240 brain volumes as a whole, each of them made up of 32 axial slices. Additionally, three‐dimensional T1‐weighted imaging (T1WI) images were taken by a magnetization‐prepared rapid gradient‐echo (MPRAGE) sequence. Detailed parameters were as follows: TR = 2530 ms, TE = 2.98 ms, FOV = 256 × 256 mm^2^, in‐plane matrix = 256 × 256, comprising a total of 192 sagittal slices at a thickness of 1 mm. Subsequently, routine MRI scanning—including T1WI, T2‐weighted imaging (T2WI) and T2‐fluid‐attenuated inversion recovery (T2‐FLAIR) sequences—was performed to exclude significant cerebral pathology.

### 2.4 Data Preprocessing

The resting‐state functional MRI (rs‐fMRI) data were preprocessed by the DPABI toolkit (http://rfmri.org/dpabi) in order to determine gBOLD‐CSF coupling. Following the exclusion of the initial 10 volumes, slice‐timing correction and skull stripping were conducted. To separate CSF signals, the data were subjected to a low‐pass filter within 0.01 to 0.1 Hz and detrended. The rs‐fMRI sequence was subjected to a thorough set of preprocessing procedures that included movement correction, low‐pass filtration (0.01–0.1 Hz), linear and quadratic detrending and Gaussian kernel smoothing with a full width at half maximum of 4 mm for evaluating the effects of gBOLD signals. Participants' head motion in maximum translation bigger than 1.5 mm or maximum rotation bigger than 1.5° was not included in subsequent studies. In this study, three BQD individuals and two HCs were excluded due to excessive head motion.

### 2.5 gBOLD‐CSF Coupling

**FIGURE 2:**
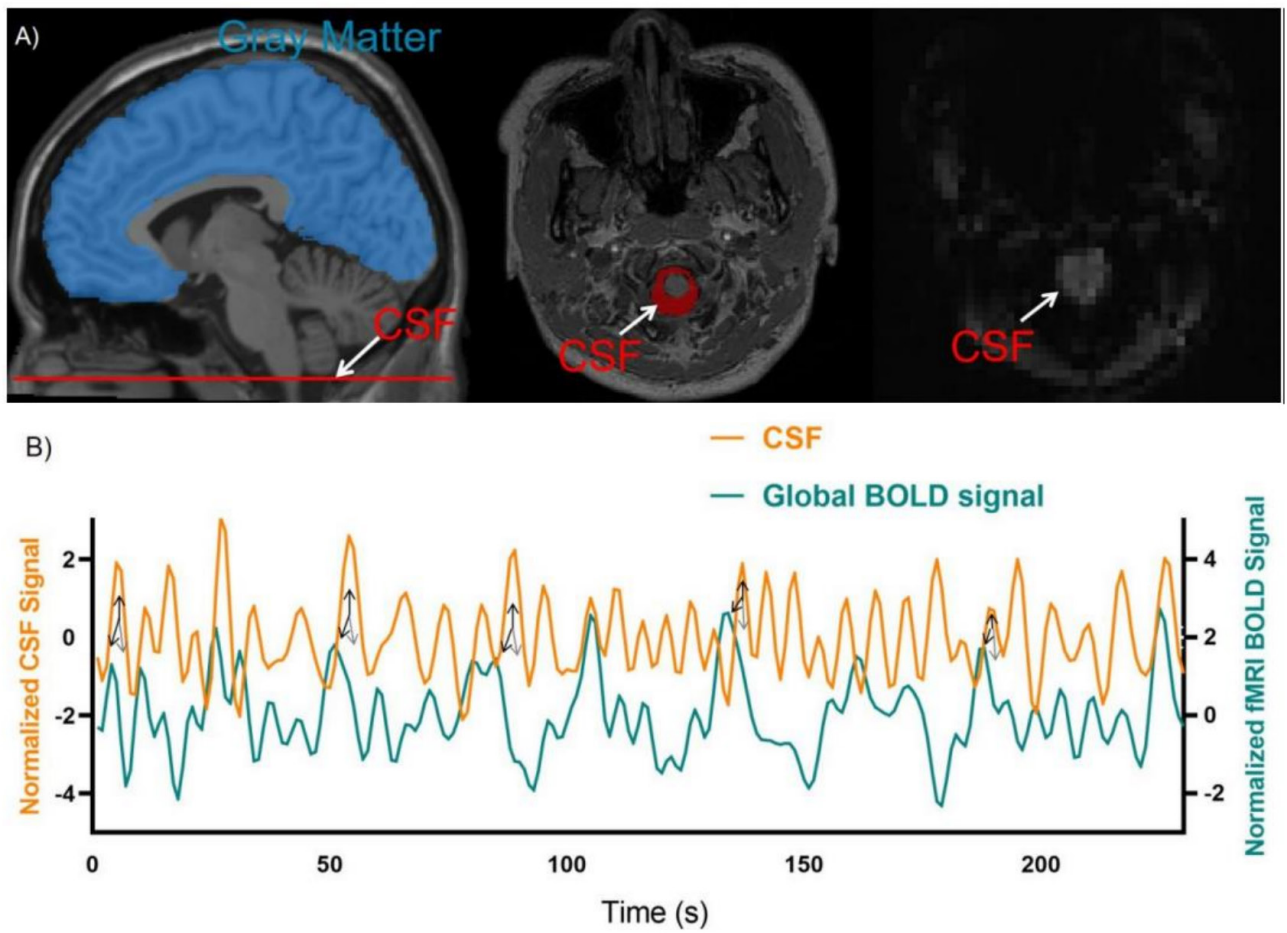
Changes of gBOLD and CSF signal. (A) The blue mask on the T1‐weighted image illustrates that gBOLD signal was derived from average data within brain's grey matter. In contrast, CSF signal was taken out from the CSF location shown in the figure, which is located in the fMRI acquisition's bottom slice. (B) A typical subject's gBOLD and CSF signal. A gBOLD positive peak (down‐pointing black arrow) typically precedes a CSF peak (up‐pointing black arrow), and a large gBOLD negative peak (shown by the grey arrow) typically follows. CSF, cerebrospinal fluid; gBOLD, global blood‐oxygen‐level‐dependent.

After preprocessing rs‐fMRI images, the whole‐brain gBOLD signals were obtained from the grey matter mask of the automatic anatomical annotation (AAL) atlas (Figure 2). The slice at the junction of the lower cerebellum and upper spinal cord/medulla oblongata was selected for CSF signal referencing (Figure 2), as this region is considered particularly sensitive to inflow effects [27]. Participants were excluded if this specific slice was not fully covered in their scans. Due to variations in FOV settings across sessions, this criterion led to a relatively high exclusion rate. For the remaining participants, the signal intensity from this slice was used to derive the average CSF time series. There were corresponding amplitude changes in the CSF and gBOLD signals. A large gBOLD positive peak typically precedes the CSF peak, and a large gBOLD negative peak typically follows (Figure 2B). The cross‐correlation was calculated for figuring out gBOLD‐CSF coupling strength with a time lag spanning between −10 and 10 s. Quantifying each participant's gBOLD‐CSF coupling strength through figuring out the negative peak correlation coefficient at approximately +4‐s delays. Furthermore, in accordance with Jiang et al. [25], the cross‐correlation between the CSF signal and the negative first‐order derivative of the gBOLD signal was computed. The correlation coefficients were compared to the values noticed after randomly matching BOLD and CSF signals from various individuals 10 000 times.

### 2.6 Statistical Analysis

Evaluating the clinical and demographic traits among the BQD and HC groups through IBM SPSS Statistics Software (version 25.0). Since most continuous variables do not adhere to a normal distribution, the differences in continuous variables between two groups were assessed via two‐sample nonparametric tests. Chi‐square test was employed for gender. Group effects on gBOLD‐CSF coupling were compared through two‐sample *t*‐tests. In the BQD group, exploratory Pearson's correlation analyses were conducted to examine the associations between gBOLD‐CSF coupling and clinical variables, including BQDS, duration, daily consumption, HAMA‐14 and HAMD‐24. To address the issue of multiple comparisons, a Bonferroni correction was applied. With five correlation analyses performed, the corrected significance threshold was set at *p* < 0.01 (0.05/5). Effect sizes (correlation coefficients *r*) are also reported to facilitate interpretation of the findings.

## 3 Results

### 3.1 Demographic and Clinical Data

Sixty‐six volunteers participated, consisting of 29 BQD individuals and 37 HCs. Age, HAMA‐14 and HAMD‐24 scores did not process distinctions within two groups (all *p* > 0.05). However, significant variations among groups were observed in sex distribution and educational level (all *p* < 0.05). Among participants with BQD, the mean duration of BQ chewing was 15.3 ± 7.4 years, and the mean BQDS score and daily BQ consumption number among the BQD individuals were recorded at 8.8 ± 2.7 and 6.1 ± 7.2, respectively. Detailed information is shown in Table 1.

**TABLE 1:** Characteristics of participants.

	BQD (*n* = 29)	HC (*n* = 37)	*p*
Gender (males/females)	13/16	5/32	0.005^a^
Age (year)	43 (36–48)	37 (27–45.5)	0.720^b^
Education (year)	9 (9–12)	16 (11.5–17)	0.000^b^
HAMA‐14 (score)	2 (0–3.5)	2 (1–4)	0.302^b^
HAMD‐24 (score)	3 (0.5–5)	4 (1–6.5)	0.309^b^
% reporting no use of alcohol	65.5	N/A	
% reporting no use of tobacco	62.1	N/A	
BQDS (score)	8.8 ± 2.7	N/A	
Daily BQ dosage (number)	6.1 ± 7.2	N/A	
Duration of BQD (year)	15.3 ± 7.4	N/A	

*Note:* Gender is shown as a number, and chi‐square tests were employed to compare groups. Continuous variables were analysed using nonparametric tests and are reported as median values with interquartile ranges. Means ± standard deviations are used to express the BQDS, BQ dosage and BQD duration values.

Abbreviations: BQ, betel quid; BQD, betel quid dependence; BQDS, Betel Quid Dependence Scale; HAMA‐14, Hamilton Anxiety Rating Scale‐14 item; HAMD‐24, Hamilton Depression Rating Scale‐24 item; HC, healthy control; N/A, not applicable.

^a^ The chi‐square test was used to figure out the *p*‐value.

^b^ An independent sample nonparametric test was employed to determine the *p*‐value.

### 3.2 Group Differences in gBOLD‐CSF Coupling

**FIGURE 3:**
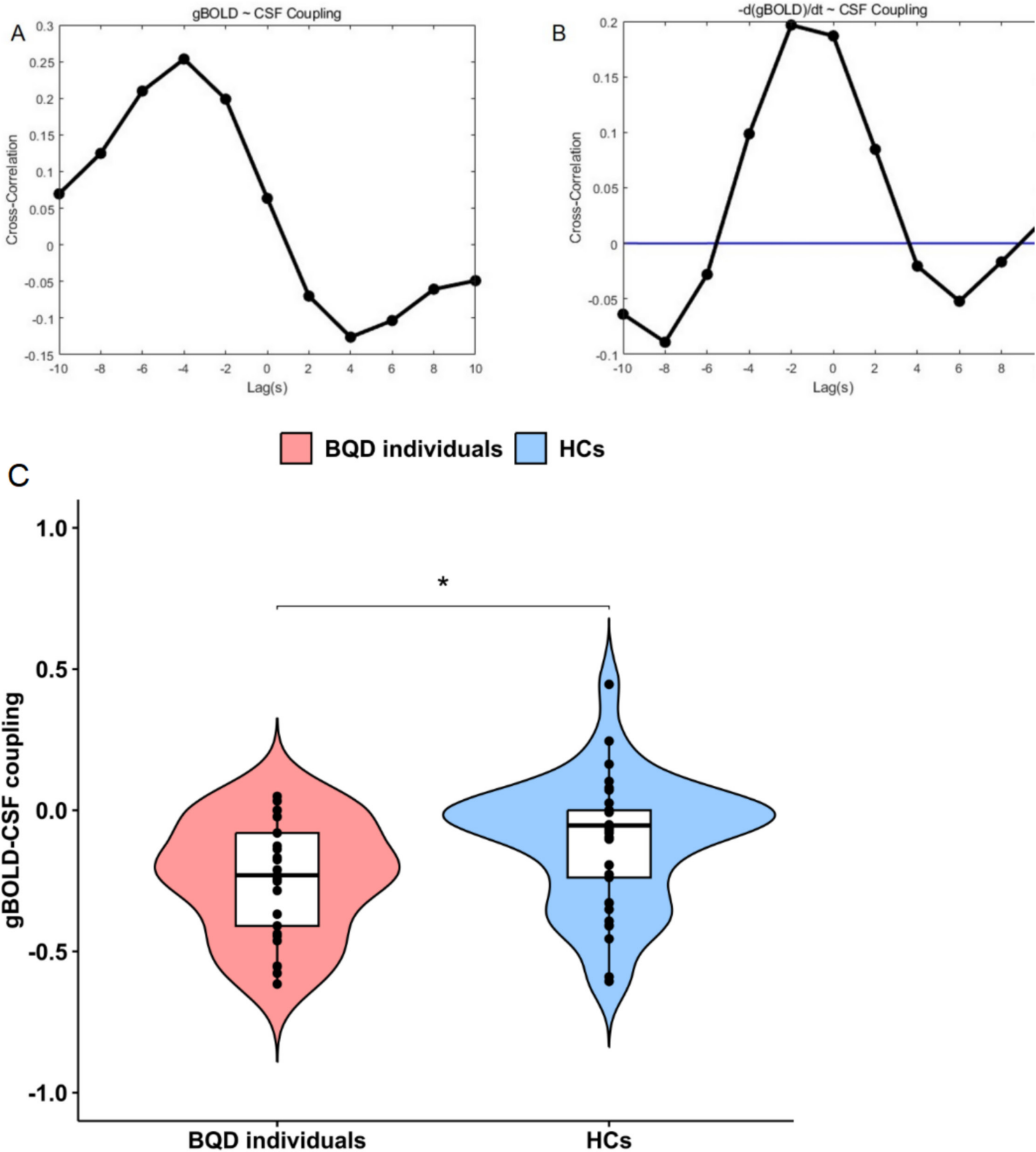
The coupling between gBOLD and CSF signals. The mean cross‐correlation between gBOLD and CSF signals across all participants is presented in Panel A. The negative derivative of the cross‐correlation between CSF and gBOLD signals is displayed in Panel B. The negative peak strength in both HCs and BQD individuals at lag +4 s is illustrated in Panel C. Abbreviations: BQD, betel quid dependence; CSF, cerebrospinal fluid; gBOLD, global blood‐oxygen‐level‐dependent; gBOLD‐CSF coupling, the coupling between global blood‐oxygen‐level‐dependent signals and cerebrospinal fluid signals; HC, healthy control; * represents *p* < 0.05.

A strong correlation between brain cortical activation and CSF flow was identified within the data that we collected, showing patterns consistent with previous findings (Figure 3) [20]. The gBOLD‐CSF cross‐correlation presented a positive peak (mean *r* = 0.2535, *p* < 0.001) at a lag of −4 s and a negative peak (mean *r* = −0.1259, *p* < 0.001) at a lag of +4 s across all participants. In addition, the cross‐correlation between the negative first‐order derivative of the gBOLD‐CSF signal exhibited a positive peak at time lags of −2 s (*r* = 0.1967, *p* < 0.001) (Figure 3). Following the approach described by Han et al. [24], the coupling strength for everyone involved was quantified using the cross‐correlated coefficient at the negative peak (+4‐s time lag). An independent sample *t*‐test initially revealed that individuals with BQD had significantly lower gBOLD‐CSF coupling than controllers (BQD = −0.240 ± 0.200, HC = −0.111 ± 0.225, *t* = −2.417, *p* = 0.019) (Figure 3C). However, after adjusting for sex, age and educational level, no significant between‐group difference in gBOLD‐CSF coupling was observed.

### 3.3 Association Between gBOLD‐CSF Coupling and Clinical Features

**FIGURE 4:**
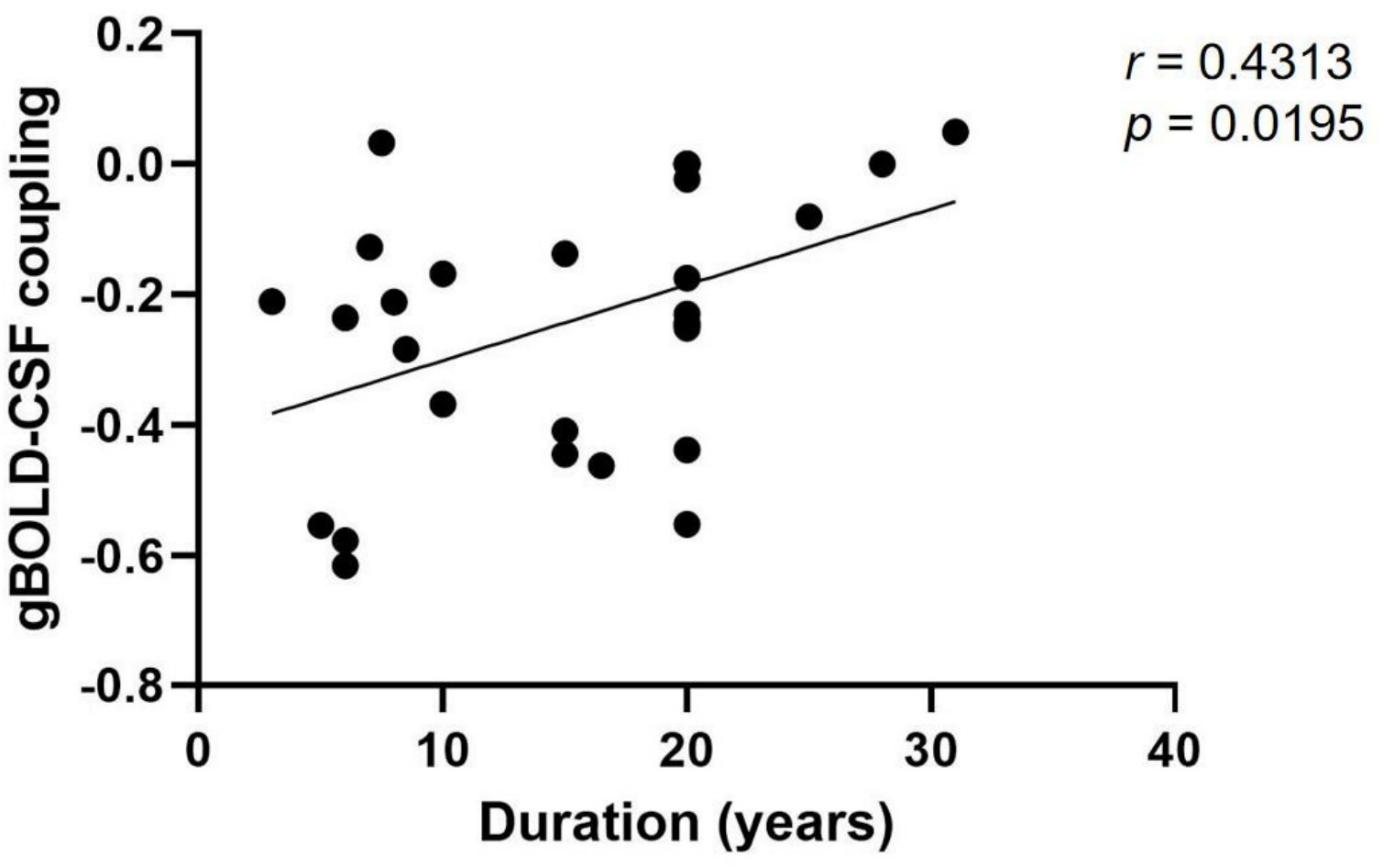
Exploratory correlation analysis between BQD duration and gBOLD‐CSF coupling (*n* = 29). Prior to multiple comparison correction, a nominally significant positive correlation was observed (*r* = 0.431, uncorrected *p* = 0.0195). After applying Bonferroni correction for five comparisons (corrected significance threshold: *p* < 0.01), this association did not reach statistical significance. BQD, betel quid dependence; gBOLD‐CSF coupling, the coupling between global blood‐oxygen‐level‐dependent signals and cerebrospinal fluid signals.

Exploratory Pearson correlation analyses were performed to examine the associations between gBOLD‐CSF coupling and clinical variables in the BQD group, including BQDS, duration of BQD, daily BQ consumption, HAMA‐14 and HAMD‐24. Among these variables, BQD duration demonstrated a nominally significant positive correlation with gBOLD‐CSF coupling (*r* = 0.431, uncorrected *p* = 0.0195; Figure 4), with a moderate effect size (*r* ^2^ = 0.186). However, this association did not remain statistically significant after Bonferroni correction for multiple comparisons (corrected significance threshold: *p* < 0.01). No significant associations were observed for BQDS, daily BQ consumption, HAMA‐14 or HAMD‐24, either before or after correction (all uncorrected *p* > 0.05).

## 4 Discussion

This work offers the first in vivo proof of glymphatic malfunction in individuals with BQD, demonstrated by significantly reduced coupling between cerebral activity and CSF inflow. Employing rs‐fMRI‐derived gBOLD‐CSF coupling, we identified a robust attenuation of neurofluid synchronization in BQD, which was further correlated with the duration of BQD. These results extend the emerging concept that neurotoxic exposure and chronic addictive behaviours may impair glymphatic dynamics.

The gBOLD‐CSF coupling index reflects spontaneous neurovascular oscillations that drive CSF inflow into the perivascular and subarachnoid spaces [20]. In this study, we examined whether individuals with BQD exhibited different coupling relationships between cortex activity and CSF flow by employing the gBOLD‐CSF coupling technique. Our findings revealed a weaker gBOLD‐CSF coupling in individuals with BQD than in HCs in the unadjusted analysis, but this difference did not remain significant after adjustment for demographic variables. This observation is similar to earlier studies on cortical glymphatic dysfunction [28, 29]. Li et al. [28] noted that individuals with severe obstructive sleep apnea demonstrated weaker gBOLD‐CSF coupling than HC participants. Furthermore, poorer cognitive performance was associated with diminished gBOLD‐CSF coupling. Zeng et al. [29] found that subjects with primary progressive aphasia exhibited lower gBOLD‐CSF coupling than HCs, suggesting glymphatic function deficits among these individuals. Additionally, they observed that clinical dementia rating and gBOLD‐CSF coupling were positively correlated. Drawing from these parallels, the reduced gBOLD‐CSF coupling observed in our cohort may suggest an alteration in glymphatic system function. Given the established role of the glymphatic system in clearing metabolic wastes such as Aβ and tau protein [30], and its proposed involvement in neurodegenerative processes [31], this finding may indicate a biologically plausible link between BQD and altered glymphatic function. However, because the present study did not directly measure the clearance rate of metabolic products or biomarkers of neuronal injury, we cannot determine whether the reduced coupling observed here reflects impaired waste clearance or contributes to neuronal injury and functional decline. These possibilities require direct validation in future studies incorporating positron emission tomography (PET) or CSF biomarker analysis, or other direct measures of glymphatic clearance and neuronal injury.

Reduced gBOLD‐CSF coupling in BQD may reflect altered glymphatic‐related brain function and may offer a potentially informative perspective on the neurobiological consequences of chronic BQ exposure. As a noninvasive MRI‐derived metric, gBOLD‐CSF coupling may warrant further investigation as a research marker for characterizing brain alterations in BQD. However, given the exploratory nature of the present findings and the lack of direct clinical or biomarker validation, its translational utility remains to be established in future longitudinal and multimodal studies.

Notably, a variety of factors influence brain activity and CSF inflow, such as arterial pulsation, vasomotion, sensory stimulation and hypertension. For instance, existing research has confirmed that changes in cerebral blood volume can modulate low‐frequency CSF activities [32]. In addition to the choroid plexus's impaired production of CSF, reduced CSF flux, AQP‐4 dysfunction and vascular factors, including compromised vascular integrity and heart pulsation, may also result in glymphatic deficits. Alterations in the gBOLD‐CSF coupling should be interpreted with these factors in consideration.

Additionally, in the BQD group, we observed a nominally significant positive correlation between BQD duration and gBOLD‐CSF coupling prior to multiple comparison correction, with a moderate effect size (*r* = 0.43). This finding tentatively suggests that prolonged BQ exposure may have cumulative effects on glymphatic function. This is consistent with our previous research that longer BQD duration correlates with altered spontaneous brain activity and cortical thickness [33, 34]. These findings indicate that long‐term BQD has a significant impact on brain structure and function. Interestingly, this correlation was observed only with BQD duration and gBOLD‐CSF coupling; BQDS failed to demonstrate such a relationship. This suggests that the duration of BQD may exert a more significant influence on glymphatic function compared to BQDS. Furthermore, no association was observed between HAMA‐14 or HAMD‐24 scores and gBOLD‐CSF coupling, implying that anxiety or depression symptoms within BQD individuals might not play a role in glymphatic dysfunction. The correlation analyses between gBOLD‐CSF coupling and clinical characteristics in the BQD group should be interpreted cautiously, as these exploratory analyses were conducted in a relatively small sample. Although the duration of BQD showed a nominal association before correction, this finding did not survive Bonferroni correction, highlighting the need for validation in larger samples.

A few limitations exist, even though this study offers insightful information about the dysregulation of glymphatic clearance in BQD. First, due to its cross‐sectional character, we are limited to identifying abnormal gBOLD‐CSF coupling in individuals with BQD and cannot directly establish a causal relationship between BQD and glymphatic dysfunction. Consequently, future research should consider employing a longitudinal study design to elucidate cause‐and‐effect linkages. Second, due to the limited sample size, group differences in sex and education level could have confounded the results. Therefore, it is essential to utilize a larger sample size in future research and to control for gender and educational level as well. Third, the small sample size of the BQD group (*n* = 29) limited the statistical power of the correlation analyses, posing a risk of Type II error and potentially leading to undetected true associations. Although we applied a Bonferroni correction to address multiple comparisons, this conservative approach may increase the risk of Type II error. Therefore, the observed association between BQD duration and gBOLD‐CSF coupling (which was significant only before correction) should be considered preliminary and hypothesis‐generating. Future large‐scale studies with preregistered primary outcomes are warranted to validate these findings while adequately controlling for multiple comparisons. Fourth, our study did not directly assess neurotoxic protein accumulation, limiting our ability to link altered coupling with molecular pathology. Future research should focus on quantifying neurotoxic molecules in both the cerebral cortex and CSF. Although the rigorously screened sample ensured the inclusion of participants without diagnosed alcohol or nicotine dependence, the use of alcohol and nicotine remains highly prevalent among individuals with BQD, and their residual effects may not have been completely eliminated. Furthermore, this is a single‐centre study, and thus the results could not be further validated by an external validation dataset. Finally, glymphatic system function in individuals with BQD was evaluated solely through single‐mode images derived from rs‐fMRI data. This limitation may obscure a robust correlation between glymphatic function and the build‐up of particular hazardous species. Emerging multimodal and machine learning approaches—as exemplified by automated body composition analysis [35] and cerebral blood flow prediction based on multimodal imaging [36]—highlight the potential of integrating diverse data modalities for complex disease assessment. By incorporating additional modalities to assess glymphatic system function, we may be able to gain a more thorough knowledge of their relationships.

## 5 Conclusion

In summary, our study reveals that individuals with BQD exhibit disrupted synchronization between cortical neural activity and CSF flow, reflecting impaired glymphatic function. These findings bridge addiction neuroscience and neurofluid physiology, providing novel mechanistic insights into how chronic arecoline exposure compromises brain homeostasis. The gBOLD‐CSF coupling metric could be used as a noninvasive imaging biomarker for detecting and monitoring glymphatic dysfunction in substance‐use disorders.

## Author Contributions

**Li Li Fu:** conceptualization, investigation, methodology, software, supervision, validation, writing – original draft, writing – review and editing. **Chao Qi Lv:** data curation, methodology, project administration, writing – review and editing. **Li Ting Liu:** data curation, methodology, project administration, writing – review and editing. **Qing Qing Fu:** data curation, methodology, project administration, writing – review and editing. **Wei Yuan Huang:** conceptualization, methodology, validation, writing – review and editing. **Yi Hao Guo:** conceptualization, methodology, validation, writing – review and editing. **Tao Liu:** conceptualization, methodology, validation, writing – review and editing. **Hui Juan Chen:** conceptualization, investigation, methodology, supervision, validation, writing – original draft, writing – review and editing. **Feng Chen:** conceptualization, investigation, methodology, supervision, validation, writing – original draft, writing – review and editing.

## Funding

This study was supported by the Key Science and Technology Project of Hainan Province (ZDYF2023SHFZ096, ZDYF2024SHFZ058); the National Nature Science Foundation of China (Grant Nos. 82160327, 82271977); the Hainan Provincial Natural Science Foundation of China (826QN0855) and the Hainan Academician Innovation Platform Fund, the Hainan Province Clinical Medical Center.

## Ethics Statement

The ethics review board at our hospital gave its approval for this study (Number 2017‐4). Prior to inclusion, each participant gives their informed consent.

## Conflicts of Interest

The authors declare no conflicts of interest.

## Data Availability

The data that support the findings of this study are available from the corresponding author upon reasonable request.
